# Use of Platelet‐Rich Platelet Aggregates (PRFs) in Soft Tissue Repair in Periodontics: A Systematic Review

**DOI:** 10.1002/cre2.70327

**Published:** 2026-04-28

**Authors:** Ilan Hudson Gomes de Santana, Ozawa Brasil de Júnior, Anderson Jara Ferreira, Katia Caetana Pereira, José Marcos Pereira Júnior, Erick Andres Alpaca Zevallos, Gustavo Ramiro Rojas Manrique, Jaime S. Gallegos Zanabria, Camila Coelho Guimarães, Ennyo Sobral Crispim da Silva

**Affiliations:** ^1^ Health Sciences Center, Undergraduate Student in Dentistry Federal University of Paraíba João Pessoa Brazil; ^2^ Health Sciences Center Federal University of Paraíba (UFPB) João Pessoa Brazil; ^3^ Catholic University of Santa María Santa María Peru; ^4^ São Leopoldo Mandic College São Paulo Brazil; ^5^ Professor of Periodontics Federal University of Paraíba João Pessoa Brazil

**Keywords:** connective tissue, gingival diseases, platelet‐rich fibrin, surgical flaps

## Abstract

**Objectives:**

To evaluate, through a systematic review, the clinical effectiveness of platelet‐rich fibrin (PRF) in periodontal soft tissue repair compared with conventional regenerative techniques.

**Material and Methods:**

This systematic review followed PRISMA 2020 guidelines and was registered in PROSPERO (CRD42024617061). Searches were conducted in PubMed, Scopus, Web of Science, ScienceDirect, and LILACS, complemented by gray literature sources. Randomized clinical trials evaluating PRF or its derivatives in periodontal soft tissue repair were included. From 996 identified records, 28 studies met the eligibility criteria and were included in the qualitative analysis.

**Results:**

PRF demonstrated clinical outcomes comparable to conventional techniques such as connective tissue grafts and other biomaterials for root coverage and periodontal regeneration. In addition, PRF was associated with reduced surgical morbidity, lower postoperative discomfort, and shorter surgical time.

**Conclusions:**

PRF represents a promising autologous biomaterial for periodontal soft tissue repair, providing clinical outcomes similar to conventional grafting techniques with lower morbidity. Further well‐designed clinical trials with standardized protocols and long‐term follow‐up are required.

## 1 Introduction

Modern periodontics faces the challenge of restoring not only periodontal health, but also the esthetics and function of oral soft tissues, especially in cases of gingival recession and peri‐implant defects (Carrera et al. 2023; Sabri et al. 2023). The predictability of periodontal plastic surgery procedures is traditionally associated with the use of autogenous grafts, such as subepithelial connective tissue grafting (CTG), considered the gold standard for volume augmentation and root coverage (Azadi et al. 2024). Despite their proven efficacy, these techniques have significant disadvantages, including donor site morbidity, longer surgical time, postoperative pain, and limitations related to the amount of tissue available, factors that can negatively impact patient acceptance (Abu‐Ta'a 2023; Albatal et al. 2023; İzol and Üner 2019; Aroca et al. 2013).

In this context, autologous biomaterials of blood origin have been studied as therapeutic alternatives or adjuvants (Miron et al. 2017). Among them, platelet‐rich fibrin (PRF) stands out for being a concentrate of platelets and leukocytes obtained without the addition of anticoagulants or biochemical agents, resulting in a three‐dimensional fibrin matrix capable of gradually releasing growth factors such as PDGF, TGF‐β, and VEGF. This sustained release promotes angiogenesis, accelerated healing, inflammatory modulation, and tissue regeneration (Sherif et al. 2025). In addition, PRF is low cost, simple to obtain, and free of immunological risks, as it is an autologous product (Mohan et al. 2019).

Several variations of PRF have been developed over the past few years, including L‐PRF (leukocyte‐ and PRF), A‐PRF (advanced PRF), i‐PRF (injectable PRF), as well as more recent protocols such as concentrated growth factor (CGF), horizontal PRF (H‐PRF), and titanium‐prepared PRF (T‐PRF). These variations differ mainly in centrifugation parameters, tube type used, and fibrin matrix architecture, directly influencing growth factor release, cell density, and the biological behavior of the biomaterial (Sindhusha and Ramamurthy 2023).

Although PRF has shown promising results, there is still controversy regarding its effectiveness in comparison with established techniques, such as CTG, collagen membranes, and enamel matrix derivatives (EMD) (Acerra et al. 2025). The methodological heterogeneity among clinical studies, the different preparation protocols, and the variability in the outcomes analyzed make it difficult to draw definitive conclusions about its clinical superiority or equivalence.

Recently, thematic publications dedicated to autologous platelet concentrates have consolidated clinical and biological evidence on the use of PRF in periodontics and implantology, including applications in covering gingival recessions, regenerating intraosseous defects, and treating furcation involvement. These studies reinforce the potential of PRF as a regenerative biomaterial, while highlighting the need for standardization of clinical and methodological protocols. Given this scenario, this study aims to conduct a systematic literature review on the use of platelet aggregates (PRF, L‐PRF (Leukocyte‐rich PRF), A‐PRF (Advanced PRF), and i‐PRF (injectable PRF)) in the repair of periodontal soft tissues, seeking to identify their clinical benefits, limitations, and possible implications for evidence‐based dental practice.

## 2 Materials and Methods

### 2.1 Compliance and Registration

This systematic review was conducted in accordance with the Preferred Reporting Items for Systematic Reviews and Meta‐Analyses (PRISMA 2020) guidelines (Rethlefsen and Page 2022) and the methodology established by the Cochrane Collaboration, aiming to ensure transparency, reproducibility, and methodological quality. Before the data collection phase began, the protocol was registered in the International Prospective Register of Systematic Reviews (PROSPERO) (Page et al. 2018), under number CRD42024617061, ensuring the traceability of the process and preventing selective reporting bias.

### 2.2 Formulation of the Research Question

The research question was structured based on the PICO model, which allows the question to be broken down into four fundamental elements. The target population comprised adult patients (≥ 18 years) with periodontal soft tissue repair needs, such as treatment of gingival recessions, root coverage, or periodontal and peri‐implant surgical interventions, considering different degrees of severity and demographic profiles. The intervention of interest was the clinical application of PRF, including its different variations (A‐PRF, L‐PRF, i‐PRF), used as an adjuvant in periodontal procedures. The comparator included conventional periodontal treatment approaches without the use of PRF or the use of other regenerative biomaterials. The primary outcomes were periodontal clinical parameters, such as reduction in probing depth (PD), clinical attachment gain, and change in radiographic bone level, while the secondary outcomes included healing rates, bleeding on probing indices, and the occurrence of postoperative complications. This structure aimed to ensure clarity in the selection and interpretation of evidence, enabling the accurate identification of the intervention's effectiveness compared to existing therapeutic alternatives.

### 2.3 Eligibility Criteria

The inclusion criteria were designed to select only high‐quality evidence with clinical relevance. Studies with an experimental design were eligible, particularly randomized controlled trials, non‐randomized controlled studies, and split‐mouth clinical trials, provided they were conducted in humans. Inclusion was restricted to studies published in English, Portuguese, or Spanish, with no initial time limitation, in order to include both pioneering studies and recent research. To be included, studies had to report, quantitatively and with clear metrics, at least one of the defined outcomes.

The following were excluded: case reports and series; narrative or systematic reviews; in vitro studies or studies using animal models; studies with samples composed of individuals with systemic or drug‐related conditions that could interfere with bone and periodontal metabolism (e.g., osteoporosis, chronic use of corticosteroids, bisphosphonates); studies with incomplete or unavailable data or with insufficiently described methodology to allow critical evaluation.

### 2.4 Search Strategy

The bibliographic search was planned in a broad and sensitive manner, covering multiple electronic databases: PubMed/MEDLINE, Scopus, Web of Science, LILACS, and ScienceDirect, with the intention of capturing all relevant publications and reducing omission biases (as shown in Table 1). The gray literature was explored through the Google Scholar and OpenGrey platforms, covering theses, dissertations, and conference proceedings.

**Table 1:** Strategies used for database searches.

Base	Strategy	*n*
PubMed	(“Platelet Rich Fibrin” OR “PRF” OR “Platelet‐Rich Fibrin”) AND (“Gingival Recession” OR “Root Coverage” OR “Periodontal Plastic Surgery” OR “Soft Tissue Repair”)	228
Scopus	((“Leukocyte‐Platelet Rich Fibrin” OR “L‐PRF” OR “Platelet‐Rich Fibrin”) AND (“Gingival Recession” OR “Root Coverage” OR “Periodontal Plastic Surgery” OR “Soft Tissue Repair”))	261
LILACS	(“Leukocyte‐Platelet Rich Fibrin” OR “L‐PRF” OR “Platelet‐Rich Fibrin”) AND tw:(“Gingival Recession” OR “Root Coverage” OR “Periodontal Plastic Surgery” OR “Soft Tissue Repair”)	25
Web of Science	(“Leukocyte‐Platelet Rich Fibrin” OR “L‐PRF” OR “Platelet‐Rich Fibrin”) AND (“Gingival Recession” OR “Root Coverage” OR “Periodontal Plastic Surgery” OR “Soft Tissue Repair”)	246
Science Direct	(“Leukocyte‐Platelet Rich Fibrin” OR “L‐PRF” OR “Platelet‐Rich Fibrin”) AND (“Gingival Recession” OR “Root Coverage” OR “Periodontal Plastic Surgery” OR “Soft Tissue Repair”)	236
Total duplicates	259	

*Source*: Own work.

Each strategy was adapted to the specific indexing and operators of each database, combining controlled descriptors (Medical Subject Headings—MeSH) and free terms, connected by Boolean operators (AND, OR) and applying truncations when necessary. In the PubMed database, for example, the following expression was used:

### 2.5 Selection of Studies

The selection process was carried out in two independent stages, based on the principles of reproducibility and minimization of bias. Initially, two reviewers screened titles and abstracts, excluding studies that clearly did not meet the established criteria. Next, potentially eligible articles were obtained in full text and evaluated in their entirety. Any disagreement between reviewers was discussed until consensus was reached, and in case of deadlock, a third evaluator decided on inclusion. The entire process was documented and summarized in a PRISMA flowchart, highlighting the stages of identification, screening, eligibility, and final inclusion.

### 2.6 Data Extraction

The information was extracted independently by two reviewers using a standardized, pre‐tested form. Data were collected on: author and year of publication; country where the study was conducted; sample characteristics (number of participants, age group, gender, periodontal diagnosis); type and protocol of intervention; comparator used; methods of measuring outcomes; follow‐up time; and quantitative and qualitative results. Whenever possible, exact numerical values (mean, standard deviation, confidence interval, *p*‐values) were extracted to allow for subsequent statistical synthesis.

## 3 Results

**Figure 1:**
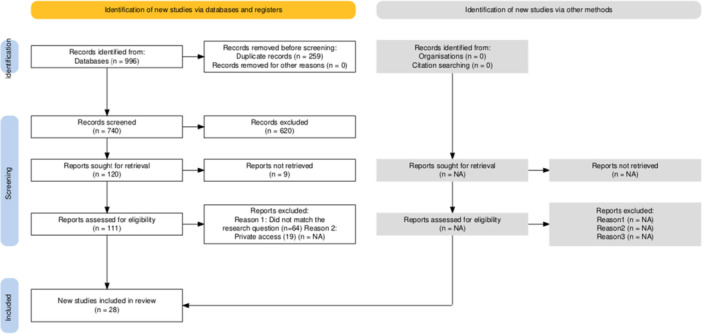
Bibliographic search flowchart, adapted from PRISMA 2020. *Source*: Own work.

The search strategy in electronic databases resulted in a total of 996 records (PubMed = 228, Scopus = 261, Web of Science = 246, ScienceDirect = 236, LILACS = 25), to which additional references retrieved from gray literature were added. After removing 259 duplicates, 737 records were screened for titles and abstracts. Of these, most were excluded because they did not meet the eligibility criteria, leaving 112 articles to be read in full. After complete evaluation, 28 studies met all methodological requirements and were included in the qualitative synthesis of this systematic review. The PRISMA 2020 flowchart (Figure 1) illustrates in detail each step of the process of identification, screening, eligibility, and final inclusion of studies.

**Figure 2:**
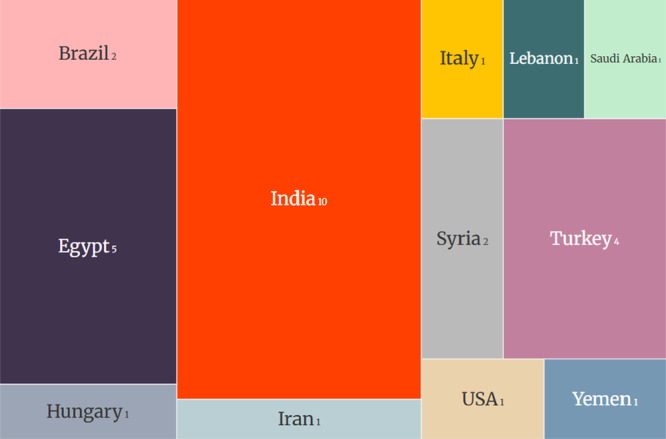
Countries of origin of the included studies and number of studies per country. *Source*: Own work.

The included studies were published between 2014 and 2025 and conducted in several countries, such as India, Egypt, Turkey, Brazil, Syria, Iran, Hungary, Italy, Lebanon, Saudi Arabia, and the United States, as shown in Figure 2. The predominant design was randomized clinical trials, including both split‐mouth and parallel studies, some in pilot format. The number of participants varied considerably, from small samples with fewer than 10 patients to studies with more than 80 treated sites.

In general, the studies evaluated the efficacy of PRF and its variations (L‐PRF, A‐PRF, H‐PRF, i‐PRF) in periodontal and peri‐implant procedures, with conventional techniques such as subepithelial CTG, collagen membranes, EMD, free gingival grafts (FGG), hyaluronic acid (HA), and flap‐only surgeries as the main comparators.

**Figure 3:**
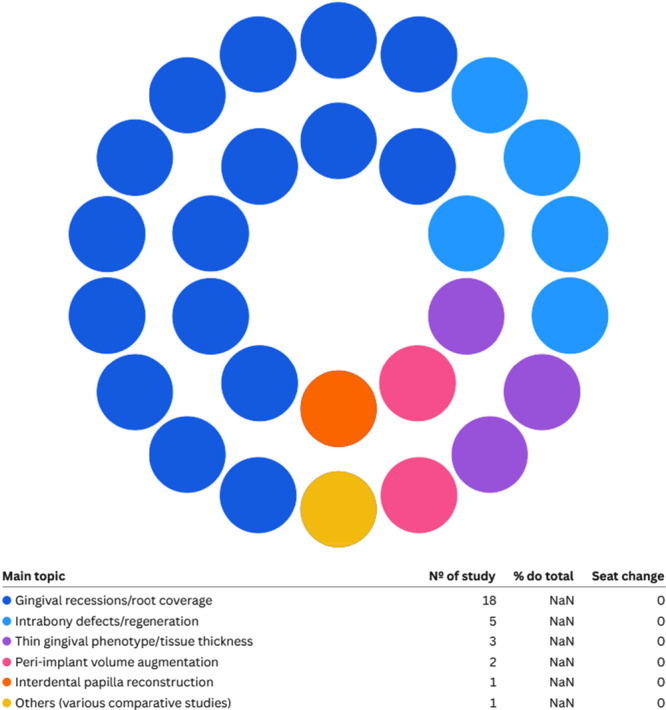
Main approaches of the studies and their respective values. *Source*: Own work.

The most frequently evaluated clinical outcomes (Figure 3) included reduction in PD, clinical attachment gain, increase in keratinized tissue width (KTW) and thickness, percentage of root coverage, healing and esthetic parameters, as well as subjective measures such as pain, discomfort, and patient satisfaction.

The summary of the results (Table 1) showed that, in gingival recessions, PRF presented clinical performance similar to CTG in terms of root coverage and clinical attachment gain, but with important advantages related to reduced postoperative morbidity, shorter surgical time, and no need for a second donor site. Comparative trials with EMD and HA also showed no statistically significant differences, reinforcing the possibility of considering PRF as a viable and lower‐cost alternative. In some studies, the combined use of PRF with other biomaterials, such as ascorbic acid or nano‐hydroxyapatite, showed additional gains in filling intraosseous defects and periodontal regeneration.

In the context of increasing peri‐implant tissue volume, PRF provided a significant increase in keratinized mucosa, although autogenous grafts still showed superior results in terms of absolute tissue gain. The use of injectable i‐PRF has shown potential for increasing gingival thickness (GT) in individuals with a thin phenotype, with results comparable to HA.

**Figure 4:**
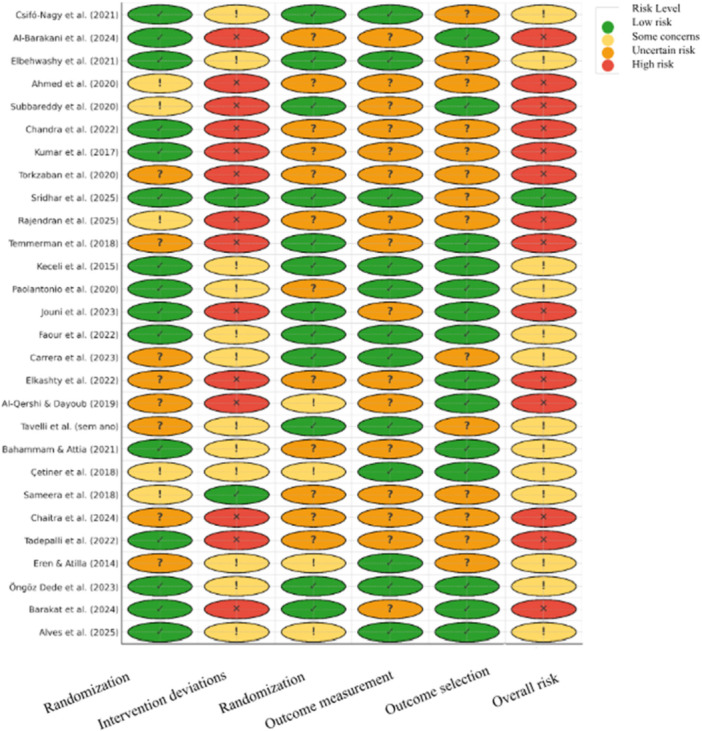
Risk of bias analysis according to the Cochrane Risk of Bias 2.0 (RoB 2) tool. *Source*: Adapted from RoB 2.

Methodological quality was assessed independently by two reviewers. For randomized clinical trials, the Cochrane Risk of Bias 2.0 (RoB 2) tool was used, which examines five critical domains: random sequence generation, allocation concealment, blinding of participants and assessors, incomplete outcome data, and selective reporting of results. For non‐randomized studies, the ROBINS‐I instrument would be adopted, which considers potential biases before, during, and after the intervention; however, there were no studies with this type of methodology. The results of this assessment are presented in Figure 4 for ease of visualization.

## 4 Discussion

This systematic review brought together 28 randomized clinical trials evaluating the use of PRF in different periodontal and peri‐implant contexts. Overall, the results show that PRF, in its various forms, offers clinical results similar to those obtained with subepithelial CTG and other biomaterials, but with less morbidity and greater comfort for patients.

Most studies focused on the treatment of gingival recessions. Eren and Atilla (2014) observed that the mean root coverage was similar between CAF + PRF (92.7%) and CAF + CTG (94.2%), with complete coverage in 72.7% and 77.3% of sites, respectively. Both groups increased KTW and GT, although PRF reduced PD slightly more (1.09 mm vs. 1.45 mm; *p* = 0.017).

Keceli et al. (2015) showed that the combination of CAF + CTG + PRF was more effective in tissue thickness gain (TT: 1.96 ± 0.34 mm vs. 1.55 ± 0.37 mm; *p* < 0.05) compared to CAF + CTG alone, suggesting an additive effect of PRF in sites with a thin phenotype, although there were no differences in gingival margin position (VR) or clinical attachment level (CAL). Kumar et al. (2017) compared CAF alone, CAF + CTG, and CAF + PRF. After 6 months, vertical gingival gain was similar in PRF and CTG (1.26 mm each), both significantly higher than CAF alone (0.53 mm). Complete root coverage was higher in PRF (60%) than in CTG (20%), indicating its replacement potential.

Chandra et al. (2022), using the Pouch and Tunnel technique, reported that PRF and CTG were equally effective in clinical attachment gains, but PRF promoted less pain on D1 and significant increases in KTW (+ 1.45 mm in 6 months) and GTMB (+ 1.0 mm in 1 month). Carrera et al. (2023), in 16 months of follow‐up, also found similar esthetics between the groups (RES = 7.0), but greater patient satisfaction with CTG (VAS = 9.65 vs. 8.60), although PRF reduced operating time. Subbareddy et al. (2020) reported that PRF achieved significant gains in CAL (3.73 ± 1.92 mm) compared to CTG (2.58 ± 0.92 mm; *p* = 0.011), in addition to greater GT (1.09 ± 0.27 mm vs. 0.99 ± 0.08 mm; *p* = 0.020). Jouni et al. (2023) demonstrated that CTG was superior in GT and KTW, but PRF had a better healing index (WHI), showing more favorable initial repair.

Other studies have explored different surgical techniques. Torkzaban et al. (2023) showed that MARF + PRF increased gingival width and thickness and reduced wound retraction (30.9% vs. 51.1%; *p* = 0.003) without increasing postoperative pain. Chaitra et al. (2024) compared PRF and amniotic membrane over 9 months, finding similar results in ALO gains and gingival margin position, confirming PRF as a viable autologous alternative. Al‐Barakani et al. (2024) tested the Pinhole Surgical Technique associated with A‐PRF versus collagen membrane, reporting similar results in pain (NRS), KTW, and GT at 3 months, with a favorable trend toward PRF.

Tadepalli et al. (2022) compared A‐PRF and L‐PRF associated with CAF, both effective in improving clinical parameters at 6 months. At 3 months, L‐PRF showed greater KTW (3.67 ± 0.49 mm vs. 3.13 ± 0.64 mm; *p* = 0.016), but this difference disappeared at 6 months. Sridhar et al. (2025) found that H‐PRF provided greater complete root coverage (64.3% vs. 33.3%; *p* = 0.005) and greater GT (1.09 ± 0.14 mm vs. 1.02 ± 0.15 mm; *p* = 0.025) compared to A‐PRF + , reinforcing the importance of the centrifugation protocol. Öngöz Dede et al. (2023) also compared CGF, A‐PRF, and isolated CAF in RT1 recessions, reporting similar gains in root coverage, although autologous concentrates favored greater tissue thickness.

Elbehwashy et al. (2021) reported that OFD + AA/PRF achieved greater PD reduction (from 8.25 ± 1.50 mm to 4.05 ± 1.34 mm) and greater CAL gain than PRF alone. Bahammam and Attia (2021) found that PRF+nano‐HA achieved greater CAL gains (4.5 ± 1.42 mm) and better radiographic filling (2.31 ± 0.75 mm) than PRF alone. Paolantonio et al. (2020) confirmed the non‐inferiority of L‐PRF + ABG compared to EMD + ABG in unconfined intraosseous defects, with similar final CAL values (6.68 ± 1.02 mm vs. 6.93 ± 1.05 mm; *p* = 0.37). Csifó‐Nagy et al. (2021), comparing A‐PRF+ and EMD, reported similar clinical results but highlighted low statistical power (~11%) and the need to include groups without biomaterials.

Temmerman et al. (2018) showed that FGG achieved greater absolute keratinized mucosa gain (7.3 ± 1.2 mm vs. 6.0 ± 0.8 mm), but PRF was associated with less retraction (23.6% vs. 32.1%) and greater comfort. Elkashty et al. (2022) found no significant differences between SCTG and PRF for peri‐implant GT, reinforcing the potential of PRF as a less invasive alternative. Barakat et al. (2024) observed similar papillary height gains between multilayer A‐PRF and CTG (2.25 ± 0.97 mm vs. 1.86 ± 0.7 mm), but the PRF group required fewer analgesics (8 ± 3.08 vs. 11.75 ± 3.51; *p* = 0.003).

Faour et al. (2022) reported that multiple injections of i‐PRF increased GT by ~1.02 mm in 3 months, results comparable to HA (1.05 mm). Patra et al. (2025) observed similar reductions in PD after RAR in both groups (4.7 ± 1.5 mm vs. 4.56 ± 1.42 mm), with no additional benefits from i‐PRF, but confirming its clinical safety. The findings of this review indicate that PRF is a safe, low‐cost autologous biomaterial with results equivalent to conventional materials in different clinical contexts. It has clear advantages such as lower surgical morbidity, absence of a donor site, greater comfort, and reduced operating times. However, methodological limitations were evident, including small sample sizes, heterogeneity in preparation protocols, and different follow‐up times. As highlighted by Csifó‐Nagy et al. (2021; Saleh et al. 2024; Chambrone et al. 2022), the absence of groups without biomaterials makes it difficult to assess the isolated effect of PRF.

Consistently, studies have shown that PRF improves postoperative comfort, reduces pain, and accelerates initial healing, in addition to presenting satisfactory esthetic results, often similar to those obtained with connective tissue grafts. However, methodological heterogeneity, the diversity of surgical techniques employed, and limited follow‐up time in some trials make it difficult to draw a definitive conclusion about the superiority of the technique. Nevertheless, the evidence gathered suggests that PRF can be considered a promising and clinically effective biomaterial with relevant benefits for the patient.

Although dozens of randomized clinical trials have evaluated the use of PRF in different periodontal contexts, including regeneration of intraosseous and furcation defects, the number of studies included in this review was reduced due to the strict eligibility criteria adopted. Only randomized clinical trials that specifically evaluated the repair of periodontal soft tissues, with clearly defined clinical outcomes and adequate comparators, were included. This methodological approach, while increasing the internal robustness of the analysis, limits the scope of the results and should be considered a limitation of the present study.

## 5 Conclusion

PRF has proven to be an effective alternative to traditional grafts in periodontal surgery. Its main advantage is that it eliminates the need for a second surgical site, which significantly reduces patient pain and discomfort. Studies indicate that PRF has clinical results comparable to those of connective tissue grafts for root coverage and soft tissue augmentation, establishing itself as a viable treatment option with lower morbidity.

## Author Contributions

Ozawa Brasil Júnior contributed to the study's conception, data acquisition, and critical review of the intellectual content. Ilan Hudson Gomes de Santana participated in the conception and methodological design, data curation and analysis, drafting of the original manuscript, and final critical review. Anderson Jara Ferreira contributed to data acquisition, preliminary analysis, and critical review of the manuscript. Katia Caetana Pereira participated in data collection and organization, as well as critical review of the content. José Marcos Pereira Júnior contributed to data curation and methodological review of the study. Erick Andres Alpaca Zevallos contributed to data analysis and interpretation and critical review of the manuscript. Gustavo Ramiro Rojas Manrique participated in data analysis and critical intellectual review. Jaime S. Gallegos Zanabria contributed to the interpretation of the results and critical review of the scientific content. Camila Coelho Guimarães participated in data acquisition and final review of the manuscript. Ennyo Sobral Crispim da Silva contributed to data analysis and critical review of the manuscript.

## Funding

The authors received no specific funding for this work.

## Ethics Statement

As this was a systematic review, it was not necessary to obtain approval from the research ethics committee to carry out this study.

## Conflicts of Interest

All authors declare no conflicts of interest.

## Supporting information


**Table 1:** Summary of results obtained.

## Data Availability

The authors have nothing to report.
