# Supplementary material for: Use of Platelet‐Rich Platelet Aggregates (PRFs) in Soft Tissue Repair in Periodontics: A Systematic Review
Source: Clin Exp Dent Res. 2026 Apr 28;12(2):e70327. doi: 10.1002/cre2.70327 (PMC13122118; doi:10.1002/cre2.70327)
Supplement: Supplementary file 1 — Table 1: Summary of results obtained. [file CRE2-12-e70327-s001.docx]

Table 1. Summary of results obtained

| **Author(s)** | **Title** | **Year of publication** | **Country of origin** | **Study Type** | **Material used** | **Post-processing: Details of the protocol after the procedure?** | **Patient follow-up time?** | **Tooth involved/region involved** | **Location (maxilla/mandible)** | **Comparators: Type of material (if applicable)** | **Clinical Performance (Yes/No. If yes, how was it evaluated?)** | **Variables evaluated as outcome and results (mean ± standard deviation)** | **Main results comparing between groups?** | **Main conclusions of the authors?** |
| --- | --- | --- | --- | --- | --- | --- | --- | --- | --- | --- | --- | --- | --- | --- |
| Csifó-Nagy et al. [17] | Efficacy of a new‐generation platelet‐rich,fibrin in the treatment of periodontal intrabony,defects: a randomized clinical trial | 2021 | Hungary | Randomized, controlled, prospective clinical trial | A-PRF+ (test), EMD (control) | Intrabony defects filled and fixed with sutures for wound closure and stability. Blinded examiner. | 6 months | 30 intrabony defects in 18 patients with chronic periodontitis | Not specified | EMD (Enamel Matrix Derivative) | Yes. Probing depth (PD), gingival recession (GR), clinical attachment level (CAL), FMBS, FMPS | PD, GR, CAL at baseline and 6 months. Estimated power of 11% for GR and CAL between groups. FMBS and FMPS comparable at baseline and post-operatively; FMBS decreased after surgery. | The study may not have had the statistical power to rule out the possibility of a difference between the groups, due to the limited number of defects treated (15 in each group). | Consider the limitation of the design without a control group without biomaterials, as isolated intrabony defects can present bone fill without the addition of biomaterials. |
| Al-Barakani et al. [18] | A comparative study of the effects of advanced platelet-rich fibrin and resorbable collagen membrane in the treatment of gingival recession: a split-mouth, randomized clinical trial | 2024 | Yemen | Split-mouth, randomized, blinded (examiner) | A-PRF (test), Resorbable Collagen Membrane (RCM) (control). Both with Pinhole Surgical Technique (PST) | Assessment of the numerical rating scale (NRS) for pain on days 1, 2, 3, and 4. | 3 months | 36 Miller class I or II gingival recession sites in 18 patients (lower canines, anterior teeth, or premolars) | Mandible | Resorbable Collagen Membrane (RCM) | Yes. Plaque index (PI), clinical attachment level (CAL), keratinized tissue width (KTW), recession depth (RD), recession width (RW), gingival thickness (GT), NRS | Descriptive data were expressed as mean (SD). CAL, KTW, RD, RW, GT measured at baseline and after 3 months. | No direct summary of comparisons between groups provided in the excerpts. | To evaluate the efficacy of A-PRF combined with PST for root coverage and compare it with RCM. |
| Elbehwashy et al. [19] | Clinical and radiographic effects of ascorbic acid-augmented,platelet-rich fibrin versus platelet-rich fibrin alone in intra-osseous,defects of stage-III periodontitis patients: a randomized controlled,clinical trial | 2021 | Egypt | Randomized controlled clinical trial, parallel | PRF with ascorbic acid (AA/PRF) (test), PRF alone (control). Both with open flap debridement (OFD) | Examined at baseline, 3, and 6 months post-surgery. | 3 and 6 months | 20 intraosseous defects in 20 patients with stage III grade C periodontitis (mandibular molars) | Mandible | PRF alone | Yes. CAL, PPD, RD, FMBS, FMPS, RLDD, RDBD | CAL: Significant intragroup gain at 3 and 6 months (p < 0.001). Baseline: OFD+AA/PRF: 8.25 ± 1.50 mm; OFD+PRF: 7.45 ± 0.86 mm. 3 months: OFD+AA/PRF: 4.05 ± 1.34 mm; OFD+PRF: 3.40 ± 1.20 mm. RD: OFD+AA/PRF: significant reduction of 0.90 ± 0.50 mm (3m) and 0.80 ± 0.71 mm (6m). OFD+PRF: reduction of 0.10 ± 0.77 mm (3m), increase of 0.20 ± 0.82 mm (6m) (p < 0.05). RLDD: Significant reduction in both groups (2.29 ± 0.61 mm and 1.63 ± 0.46 mm; p < 0.05). | OFD+AA/PRF and OFD+PRF significantly improved periodontal parameters. The augmentation of PRF with AA additionally improved gingival tissue gain and radiographic defect fill. Baseline RLDD and FMBS at 6 months were significant predictors of CAL reduction (p < 0.001). | PRF, with or without AA, can significantly improve periodontal parameters. Supplementing PRF with AA may additionally enhance radiographic linear defect fill and reduce gingival recession depth. |
| Ahmed et al. [20] | Leukocyte - Platelet Rich Fibrin (L-PRF) in Combination,with Tunneling Technique in Management of Gingival,,Recession: Randomized Clinical Study | 2020 | Egypt (inferred) | Randomized clinical study | Leukocyte-Platelet Rich Fibrin (L-PRF). Combined with tunneling technique | Not specified in the excerpts. | Not specified in the excerpts. | Multiple gingival recessions in the aesthetic zone (anterior teeth or premolars), involving more than 2 adjacent teeth, Miller | Not specified | Connective Tissue Graft (CTG) | Yes. Clinical efficacy. | Not specified in the excerpts. | Not specified in the excerpts. | The objective is to compare the clinical efficacy of L-PRF and CTG in the tunneling technique for managing gingival recession. |
| Subbareddy et al. [21] | Vestibular Incision Subperiosteal Tunnel Access Technique with,Platelet‐Rich Fibrin Compared to Subepithelial Connective Tissue,Graft for the Treatment of Multiple Gingival Recessions: A Randomized,Controlled Clinical Trial | 2020 | India (inferred) | Prospective randomized comparative clinical study | PRF membrane. Combined with the VISTA (Vestibular Incision Subperiosteal Tunnel Access) technique | Measurements recorded at baseline, 3 months, and 6 months after surgery. Patients tolerated the surgery well, with no serious complications. | 3 and 6 months | Multiple gingival recessions (58 recessions in 20 patients), Miller class I (37) and class II (21) | Not specified | Subepithelial Connective Tissue Graft (SCTG) | Yes. PI, GI, PD, CAL, RD, RW, WKT, GT | CAL (6 months): Test: 3.73 ± 1.92; Control: 2.58 ± 0.92 (p=0.011*). RD (6 months): Test: 1.69 ± 1.59; Control: 1.78 ± 0.30 (p=0.000*). RW (6 months): Test: 2.27 ± 1.87; Control: 1.78 ± 0.30 (p=0.010*). WKT (6 months): Test: 2.90 ± 0.99; Control: 3.74 ± 1.00 (p=0.000*). GT (6 months): Test: 1.09 ± 0.27; Control: 0.99 ± 0.08 (p=0.020*). | Various parameters showed statistically significant differences between the test and control groups over time. | The study aims to evaluate the efficacy of these procedures in patients seeking root coverage of multiple gingival recessions. |
| Chandra et al. [22] | Comparative Evaluation of Platelet‐Rich Fibrin versus Connective Tissue,Grafting in Treatment of Gingival Recession Using Pouch and Tunnel,Technique: A Randomized Clinical Study | 2022 | India (inferred) | Randomized clinical trial | PRF (Group I), CTG (Group II). Both with the Pouch and Tunnel (P&T) technique | Phase I therapy. Re-evaluation after 1 month. | 6 months | 40 gingival recession (GR) sites in 17 patients | Not specified | Connective Tissue Graft (CTG) | Yes. PSDL, HS, PES, RC, WKG, GTMB | PSDL (Day 1): GI: 2.95 ± 0.17; GII: 5.45 ± 0.15 (Difference of 2.5, p<0.001*). WKG (Baseline vs 6 months): GI: 1.45 (<0.001); GII: 1.35 (<0.001) . GTMB (Baseline vs 1 month): GI: 1.00 (<0.001) *; GII: 0.25 (0.019). | On day 1 and at 1 month, group II (CTG) had significantly more post-surgical discomfort (PSDL) than group I (PRF). | Both techniques (PRF and CTG with P&T) were effective. Long-term multicenter studies are needed to evaluate the clinical outcome of autologous PRF compared to CTG using the P&T procedure. |
| Kumar et al.[23] | Patient‐centered Microsurgical Management of Gingival Recession using,Coronally Advanced Flap with Either Platelet‐rich Fibrin or Connective,Tissue Graft: A Comparative Analysis | 2017 | India | Randomized controlled clinical study | PRF (Group I), CTG (Group II), CAF alone (Group III). All with coronally advanced flap (CAF) and microsurgical technique | 0.2% chlorhexidine rinse. Sutures and dressings removed after 10 days. Follow-up at 3 and 6 months. Oral hygiene reinforcement. | 10 days, 3 and 6 months | 45 Miller Class I and II gingival recession defects (upper anterior teeth region) | Maxilla | CTG, CAF alone | Yes. VGR, HGR, %CRC, PCS, PES, HS | VGR (6 months): GI: 1.26 ± 0.70 mm (74.4%); GII: 1.26 ± 0.59 mm (58%); GIII: 1.06 ± 0.79 mm (53.3%). CRC (6 months): 100% CRC in 60% (GI), 20% (GII), 27% (GIII). PCS (3 months): GI: 6.93 ± 1.16; GII: 5.80 ± 1.26; GIII: 6.67 ± 0.81 (p=0.01*). HS (3 months - change): GI: -0.40; GII: -1.53; GIII: -1.13 (p=0.02*). | CAF alone and in combination with PRF or CTG are effective. PRF and CAF resulted in the highest patient comfort and aesthetic scores, with a decrease in hypersensitivity. The increase in gingival thickness was significant only in the CTG group. | A long-term multicenter randomized controlled clinical study may be necessary to evaluate the clinical outcome of autologous PRF compared to CTG and CAF alone. |
| Torkzaban et al. [24] | Effectiveness of using platelet‐rich fibrin to increase,keratinized tissue around the tooth in the modified apical,"reposition flap method: A split‐mouth, randomized",controlled trial | 2022 | Iran | Split-mouth, pilot randomized controlled clinical trial | PRF (test). Combined with modified apically repositioned flap (MARF) | 0.2% Chlorhexidine. Absorbable suture. Periodontal dressing. | 8 weeks | 10 patients with <2 mm of attached gingiva (first premolars) | Lower mandible | MARF alone (control) | Yes. Width and thickness of the gingiva, PPD, VD, postoperative pain, wound shrinkage | Postoperative pain: PRF: 3.20 ± 0.91; MARF: 3.40 ± 0.69 (p=0.295). Wound shrinkage: PRF: 30.90 ± 7.9; MARF: 51.10 ± 20.30 (p=0.003). | The use of PRF with MARF significantly increased the width and thickness of the gingiva and reduced shrinkage compared to MARF without PRF. Postoperative pain scores and VD changes were similar. | The use of PRF with the MARF method significantly increased gingival width and thickness and reduced shrinkage. Further and multicenter studies are needed. |
| Sridhar et al. [25] | Horizontal platelet-rich fibrin versus advanced platelet-rich,fibrin plus in gingival recession management | 2025 | India | Prospective, double-blind clinical trial | A-PRF+ (CAF + A-PRF+), H-PRF (CAF + H-PRF). Both with coronally advanced flap (CAF) | Analgesics (paracetamol), no brushing for 4 weeks, 0.12% chlorhexidine. Sutures removed at 2 weeks. Follow-up at 1, 3, and 6 months. | 6 months | 84 sites in 44 patients (Cairo RT 1 and RT 2 recessions, ≥2 mm deep, on anterior and premolar teeth) | Maxilla | H-PRF (Horizontal Platelet-Rich Fibrin) | Yes. RD, PPD, CAL, KTH, WAG, MRC, CRC | MRC (6 months): H-PRF: 85.51 ± 19.87%; A-PRF+: 76.33 ± 22.54% (p>0.05). CRC (6 months): H-PRF: 64.3%; A-PRF+: 33.3% (p=0.005*). Gingival Thickness (GT) (6 months): H-PRF: 1.09 ± 0.14 mm; A-PRF+: 1.02 ± 0.15 mm (p=0.025*). | The H-PRF group had a significantly higher % of CRC (p < 0.05). Gingival thickness (GT) differed significantly between groups at 6 months, favoring H-PRF (p=0.025*). There were no significant differences in KTH or WAG. | Both treatment protocols resulted in similar clinical outcomes. Suggests examining larger samples with long-term follow-up using a split-mouth design. |
| Rajendran et al. [26] | Efficacy of Platelet-Rich Fibrin in Treatment of Multiple,Adjacent Gingival Recession Defects Using Minimally Invasive,Coronally Advanced Flap and Modified Coronally Advanced Flap:,A Split-Mouth Randomized Controlled Trial | 2025 | India | Split-mouth, randomized | PRF. Combined with MICAF or MCAF | Not specified in the excerpts. | Not specified in the excerpts. | 77 recession anomalies (Cairo RT1 or 2) in 18 individuals | Not specified | MCAF+PRF vs MICAF+PRF | Yes. Root coverage | Not specified in the excerpts. | Both MICAF+PRF and MCAF+PRF were effective. MICAF+PRF is the preferred choice considering patient acceptance, minimal invasiveness, and favorable root coverage results. | PRF can serve as a viable substitute for traditional graft materials in root coverage procedures. More long-term research is needed. |
| Temmerman et al. [27] | L-­PRF for increasing the width of keratinized mucosa around,"implants: A split-­mouth, randomized, controlled pilot clinical",trial | 2018 | Not specified | Split-mouth, randomized, controlled, pilot clinical trial | L-PRF (Leukocyte-Platelet Rich Fibrin) | Daily questionnaire for pain and swelling (100 mm VAS) for 7 days. | 6 weeks | 8 patients requiring bilateral soft tissue augmentation (around implants) | Not specified | Free Gingival Graft (FGG) | Yes. Width of keratinized mucosa (KM), pain and swelling via VAS | KM gain (6 weeks): L-PRF: 6.0 ± 0.8 mm; FGG: 7.3 ± 1.2 mm. FGG had an extra gain of 1.3 ± 0.9 mm (p < 0.05). Shrinkage: L-PRF: 32.1%; FGG: 23.6%. | FGG resulted in a significantly greater gain of KM compared to L-PRF. L-PRF showed greater shrinkage of the grafted tissue. | L-PRF is useful for increasing the amount of KM and can be considered an alternative option. However, FGG is still the gold standard. More clinical trials with larger samples are mandatory. |
| Keceli et al. [28] | The Adjunctive Effect of Platelet-Rich,Fibrin to Connective Tissue Graft in the,Treatment of Buccal Recession Defects:,"Results of a Randomized, Parallel-Group",Controlled Trial | 2015 | Turkey | Randomized, controlled, parallel-group, single-masked clinical trial | PRF. Combined with coronally advanced flap (CAF) and connective tissue graft (CTG) | Cold packs, analgesics, and anti-inflammatories. No brushing for 4 weeks, use of antiseptic. Sutures removed at 2 weeks. Monthly return visits. | 6 months | 40 patients with Miller Class I or II recession defects (≥3 mm), in the anterior/premolar area, without alveolar bone loss | Incisors, canines, premolars (upper/lower) | CAF + CTG | Yes. VR, HR, PD, CAL, KTW, TT, L-AC | VR (6 months): CAF+CTG+PRF: 0.35 ± 0.52 mm; CAF+CTG: 0.65 ± 0.59 mm (p=0.070). CAL (6 months): CAF+CTG+PRF: 1.35 ± 0.52 mm; CAF+CTG: 1.70 ± 0.66 mm (p=0.064). TT (6 months): CAF+CTG+PRF: 1.96 ± 0.34 mm; CAF+CTG: 1.55 ± 0.37 mm (p=0.001*). | Both CAF+CTG+PRF and CAF+CTG were effective in reducing VR, but with no significant difference between the groups. The mean increase in tissue thickness (TT) was significantly greater (p<0.05) in patients with PRF at 1 and 6 months. | PRF can be considered an adjunct to prevent future recessions in the presence of a thin tissue phenotype, due to its effect on increasing tissue thickness. |
| Paolantonio et al. [29] | Periodontal regeneration by leukocyte and platelet-rich fibrin,with autogenous bone graft versus enamel matrix derivative with,autogenous bone graft in the treatment of periodontal intrabony,defects: A randomized non-inferiority trial | 2020 | Italy | Randomized, non-inferiority clinical trial (NIT) | L-PRF (test). Combined with autogenous bone graft (ABG) | Non-surgical therapy (SRP) 4 months before surgical treatment. | 12 months | 44 patients with stage III-IV periodontitis, with a single experimental site (intrabony defects) | Not specified | EMD (Enamel Matrix Derivative) with ABG | Yes. PPD, CAL, GR, DBL, FMBS, FMPS | PPD (12 months): L-PRF+ABG: 4.82 ± 0.86 mm; EMD+ABG: 5.18 ± 0.77 mm (p=0.10). CAL (12 months): L-PRF+ABG: 6.68 ± 1.02 mm; EMD+ABG: 6.93 ± 1.05 mm (p=0.37). GR (12 months): L-PRF+ABG: 0.96 ± 0.57 mm; EMD+ABG: 0.96 ± 0.56 mm (p=0.92). | No statistically significant differences were found between L-PRF+ABG and EMD+ABG for the clinical and radiographic parameters evaluated. | This is the first NIT comparing the clinical efficacy of L-PRF and EMD, with ABG, to treat non-contained intrabony defects. Further investigations are needed. The L-PRF+ABG combination offers negligible cost. |
| Jouni et al. [30] | Leukocytic Platelet Rich Fibrin (L-PRF) Versus Subepithelial,Connectiv Connective Tissue Gr e Tissue Graft (SC aft (SCTG) Using T G) Using Tunneling T unneling Technique in echnique in,Treatment of Gingiv eatment of Gingival Recession (Randomiz al Recession (Randomized Contr ed Controlled Clinical olled Clinical,Study) | 2023 | Lebanon, Egypt | Randomized controlled clinical trial, parallel | L-PRF (Group A). Combined with tunneling technique (TUN) | Sutures with vicryl/polypropylene for the recipient site and silk for the palate (if SCTG). | 14 days, 3 and 6 months | 20 patients (Miller Class I or II Recession (RT1)) | Not specified | Subepithelial Connective Tissue Graft (SCTG) (Group B) | Yes. PPD, CAL, RD, KTW, GT, RES, WHI | Significant improvement in mean CAL and RD for both groups. Significant difference in GT and KTW for group B, and higher RES. Significant difference in group A for WHI. | SCTG provided higher aesthetic and functional results (better color match, tissue contour, greater width of keratinized tissues). L-PRF has superior healing properties (WHI). | Both graft modalities in combination with the tunneling technique improved the gingival phenotype. L-PRF can be used as an alternative to SCTG to treat multiple gingival recession defects, eliminating the need for a second surgical site. |
| Faour et al. [31] | Evaluation of the Hyaluronic Acid Versus the,Injectable Platelet-Rich Fibrin in the,Management of the Thin Gingival Phenotype: A,Split-Mouth Randomized Controlled Clinical Trial | 2022 | Syria | Split-mouth, randomized, blinded (examiner) | Injectable i-PRF (i-PRF group) | Repeated injection 3 times with 7-day intervals. Oral hygiene instructions to minimize trauma. | 1 and 3 months | 84 sites in 14 patients with a thin gingival phenotype (GT ≤1 mm) (incisors, canines, premolars) | Anterior mandible | Hyaluronic Acid (HA) | Yes. GT, KTW, GI, BOP, PD | GT (change from baseline): i-PRF (Baseline-1m): 0.762 (p<0.001); i-PRF (Baseline-3m): 1.02 (p<0.001). HA (Baseline-1m): 0.753 (p<0.001); HA (Baseline-3m): 1.05 (p<0.001). KTW (change from baseline): i-PRF (Baseline-1m): 4.06 (p<0.001); i-PRF (Baseline-3m): 4.07 (p<0.001). HA (Baseline-1m): 4.05 (p<0.001); HA (Baseline-3m): 4.09 (p<0.001). | No statistically significant differences were observed between the two groups at any of the three evaluation times (p>0.05). | Multiple injections of i-PRF are as effective as HA injections in managing the thin gingival phenotype. |
| Carrera et al. [1] | Root coverage with platelet-rich fibrin,or connective tissue graft: a split-mouth,randomized trial | 2023 | Brazil | Split-mouth, randomized trial | PRF membranes (test). Using the tunneling technique (TT) | 0.12% chlorhexidine gluconate, amoxicillin, nimesulide, dipyrone. Sutures removed at 2 weeks. Oral hygiene reinforcement and supragingival debridement at 1, 3, 6, and 16 months. | 1, 3, 6, and 16 months | 72 teeth (36 sites per group) with multiple gingival recessions (Miller Class I or II or Cairo RT 1) in incisors, canines, and/or premolars | Maxilla | Connective tissue graft (CTG) | Yes. RD, PD, BOP, CAL, KTW, RES, VAS | RES (16 months): CTG: 7.00 (6.00–10.00); PRF: 7.00 (4.00–9.50) (no differences). Patient satisfaction (VAS, 16 months): CTG: 9.65 (7.97–10.00); PRF: 8.60 (7.67–9.62) (p < 0.05). | Patient satisfaction (VAS) was higher for the CTG group (p < 0.05). Nine patients preferred the CTG technique side compared to five who preferred the PRF side. Surgery time on the CTG side was longer than on the PRF side (not significant). | (Not provided in the excerpts). The study compares root coverage with PRF or connective tissue graft. |
| Elkashty et al. [32] | Comparison of Sub-epithelial Connective Tissue Graft and Platelet Rich Fibrin,in Peri-implant Soft Tissue Augmentation: A Randomized Clinical Split-mouth,Study | 2022 | Egypt | Split-mouth, randomized controlled | PRF (PRF group) | Prophylactic antibiotics (amoxicillin). | 6 months post-op (T1) and 3 months after prosthesis placement (T2) | 10 patients with bilateral missing teeth in the maxillary aesthetic zone and a thin gingival phenotype | Maxilla | Subepithelial Connective Tissue Graft (SCTG) | Yes. Facial gingival thickness (CBCT), Pink esthetic score (PES) | Facial gingival thickness (T0 - baseline): No significant difference between SCTG and PRF sides (p > 0.05). PES (T1 and T2): Not provided in the excerpts. | Not provided in the excerpts. | The study compares SCTG and PRF in peri-implant soft tissue augmentation. |
| Al-Qershi & Dayoub [33] | Evaluation of Platelet Rich Fibrin in the Management of,,Gingival Recession Type I/II by Miller:,A Randomized Clinical Split Mouth Study | 2020 | Syria | Split-mouth, randomized clinical trial | PRF (test). Combined with coronally advanced flap (CAF) | Pre-periodontal treatment (scaling and root planing) two weeks before surgery. | 12 months | 40 Miller type I/II gingival recession defects in 20 patients | Not specified (bilateral or contralateral) | Connective Tissue Graft (CTG) | Yes. GR, PD, CAL, WKG, RC%, CRC% | GR (12 months): Test: 0.20 ± 0.50 mm; Control: 0.05 ± 0.15 mm. RC% (12 months): Test: 95.32 ± 11.92; Control: 98.61 ± 4.37 (p=0.25). CRC% (12 months): Test: 80%; Control: 90% (p=0.60). CAL gain (12 months): Test: 2.80 ± 0.28 mm; Control: 2.49 ± 0.55 mm. WKG gain (12 months): Test: 1.31 ± 0.45 mm; Control: 1.85 ± 0.25 mm. | GR, RC%, CRC% were not significantly different between the groups (p ≥ 0.05). CAL gain in the test group was 2.83mm and 2.48mm in the control group. WKG gain in the test group was 1.31 ± 0.45mm and 1.85 ± 0.25mm in the control group. | The use of PRF+CAF in the treatment of GR is a successful and effective treatment option, and can serve as an alternative to CTGs. |
| Tavelli et al. [34] | Coronally Advanced flap vs Tunnel technique for the treatment of peri-implant,"soft tissue dehiscences with the connective tissue graft: A randomized,",,"controlled, clinical trial" | 2022 | USA | Randomized controlled clinical trial | Connective tissue graft (CTG) | Dental prophylaxis and oral hygiene 1 month before surgery. Post-operative instructions. | 12 months | 28 participants with isolated, non-molar peri-implant soft tissue dehiscences (PSTD) | Not specified (aesthetic zone) | Coronally Advanced Flap (CAF) technique vs Tunneling (TUN) technique. Both with CTG | Yes. % mean PSTD coverage, % complete PSTD coverage, KMW gain, AMW gain, MT gain, surgical time, crown-bone crest distance, IDES, VAS | Mean PSTD coverage (12 months): CAF+CTG: 90.23 ± 19.85%; TUN+CTG: 59.76 ± 34.94% (p=0.03*). CAL gain (12 months): CAF+CTG: 2.18 ± 1.05 mm; TUN+CTG: 1.21 ± 0.87 mm (p=0.01*). KMW gain (12 months): CAF+CTG: 2.57 ± 0.90 mm; TUN+CTG: 1.57 ± 1.05 mm (p=0.01*). | The CAF+CTG group achieved a significantly higher mean PSTD coverage percentage (90.23%) compared to the TUN+CTG group (59.76%) (p=0.03*). CAF+CTG also demonstrated significantly greater gains in CAL, KMW, AMW, and MT. | CAF in conjunction with CTG results in greater PSTD coverage compared to TUN + CTG. Both treatments increase mucosal thickness and keratinized tissue width. |
| Bahammam & Attia [35] | Expression of Vascular Endothelial Growth Factor Using Platelet Rich,Fibrin (PRF) and Nanohydroxyapatite (nano-HA) in Treatment of,Periodontal Intra-Bony Defects - A Randomized Controlled Trial | 2021 | Saudi Arabia | Randomized controlled clinical trial | Autologous PRF, nano-HA, combination of PRF and nano-HA, or OFD alone | Not specified in detail, other than OFD being performed. | 6 months | 60 patients (one site/patient) with intrabony defects (IBD) and chronic periodontitis (interproximal IBDs ≥3 mm) | Not specified | nano-HA alone, combination of PRF and nano-HA, OFD alone | Yes. PD, GI, CAL, bone density, IBD fill, VEGF concentration | CAL (6 months): Group I (PRF): 4.5 ± 1.42; GII (nano-HA): 4.9 ± 1.48; GIII (PRF+nano-HA): 3.1 ± 1.27; GIV (OFD): 3.7 ± 2.35. Bone density (6 months): GIII (PRF+nano-HA): 62.82 ± 24.6 (from 42.8 ± 14.6) (p=0.011*). IBD fill (6 months): GIII (PRF+nano-HA): 2.31 ± 0.75 mm (highest). | The use of PRF with nano-HA was a successful regenerative periodontal therapy for managing periodontal IBDs, unlike the use of PRF alone. Group III (PRF + nano-HA) showed the highest percentage change in CAL (40.38%). | The use of PRF with nano-HA is effective in regenerative periodontal therapy in the management of periodontal IBDs compared to the use of PRF alone. The increase in VEGF concentrations in all groups confirmed its role in angiogenesis and osteogenesis in the early stages of bone defect healing. |
| Çetiner et al. [36] | Efficiency of platelet-rich plasma on,acellular dermal matrix application with,coronally advanced flap in the treatment of,multiple adjacent gingival recessions: A,randomized controlled clinical trial | 2018 | Turkey | Split-mouth, controlled, blinded (examiner and patient) | PRP (Platelet-Rich Plasma). Combined with coronally advanced flap (CAF) and acellular dermal matrix (ADM) | Not specified in the excerpts. | 12 months | 84 teeth (42 in each group) with multiple adjacent gingival recessions (MAGRs) Miller Class I or II (≥3 mm) in non-molar teeth | Maxillary and mandibular (50% each) | CAF + ADM alone | Yes. GRD, GRW, CAL, WKT | GRD (12 months): CAF+ADM+PRP: 0.8 ± 0.1 mm; CAF+ADM: 1.1 ± 0.4 mm (p<0.05*). GRW (12 months): CAF+ADM+PRP: 0.8 ± 0.1 mm; CAF+ADM: 1.1 ± 0.4 mm (p<0.05*). CAL (12 months): CAF+ADM+PRP: 1.2 ± 0.1 mm; CAF+ADM: 1.4 ± 0.2 mm (p<0.05*). | The addition of PRP to CAF+ADM significantly improved clinical outcomes regarding the reduction of GRD, GRW, and CAL at 12 months. | The addition of PRP to CAF+ADM in the treatment of MAGRs significantly improved clinical results, according to the 1-year follow-up findings. |
| Sameera et al. [37] | Comparison of two surgical techniques in the,treatment of multiple gingival recessions,sandwiched with a combination of A-PRF,and L-PRF | 2018 | India (inferred) | Split-mouth, double-blind, randomized | Combination of A-PRF and L-PRF membranes | Oral prophylaxis and oral hygiene instructions before surgery. Patients returned 15 days after prophylaxis for the surgical procedure. | 3 and 6 months | 96 sites in 16 patients with multiple Miller Class I and II gingival recessions (≥1 mm) on adjacent teeth | Maxilla | Semilunar vestibular incision technique (Group A) vs Pouch and Tunnel technique (Group B). Both with A-PRF and L-PRF | Yes. RD, RW, CAL, KT | RD (6 months): A: 0.1875 ± 0.3779; B: 1.1875 ± 0.417 (P<0.05*). RW (6 months): A: 1.0 ± 0.3779; B: 2.0625 ± 0.417 (P<0.05*). CAL (6 months): A: 1.5 ± 0.377; B: 2.5625 ± 0.417 (P<0.05*). KT (6 months): A: 4.0625 ± 0.572; B: 2.075 ± 0.572 (P<0.05*). | The reduction of RD and RW and the gain in CAL and KT were statistically more significant in Group A (semilunar vestibular incision) than in Group B (Pouch and Tunnel). | The study aims to evaluate the advantages of the combination of L-PRF and A-PRF membranes by comparing the pouch and tunnel and semilunar vestibular incision techniques for the treatment of multiple gingival recessions. |
| Chaitra et al. [38] | Amniotic membrane versus platelet-rich fibrin in treatment of gingival,recession- a randomized control trial | 2024 | India | Randomized controlled clinical trial, split-mouth | Platelet-rich fibrin (PRF) membrane (Group A) | Not specified in the excerpts. | 3, 6, and 9 months | 32 Miller Class I gingival recession sites in 16 patients with chronic periodontitis | Both arches | Amniotic Membrane (Group B) | Yes. PI, GI, GBI, gingival sulcus depth, RAL, GML | RAL (9 months): A: 9.00 ± 1.75; B: 10.00 ± 2 (p < 0.001). GML (9 months): A: 8.00 ± 2; B: 8.500 ± 3 (p < 0.001). PI, GI, GBI (Baseline, 3, 6, 9 months): Statistically significant difference (p < 0.05). | RAL gain in both groups was statistically and clinically significant, comparable to other studies. | PRF and amniotic membrane in the treatment of gingival recession are evaluated. |
| Tadepalli et al. [39] | Comparative Evaluation of Clinical Efficacy,of Leukocyte-Rich Platelet-Rich Fibrin with,Advanced Platelet-Rich Fibrin in Management,of Gingival Recession Defects: A Randomized,Controlled Trial | 2022 | India | Randomized clinical trial | L-PRF (CAF + L-PRF) or A-PRF (CAF + A-PRF). Both with coronally advanced flap (CAF) | Not specified in the excerpts. | 6 months | 30 recession sites (15 per group) in individuals with Miller Class I and II recessions (≥2 mm) on anterior and premolar teeth | Maxilla | CAF + A-PRF vs CAF + L-PRF | Yes. RH, RW, PPD, CAL, KTH, WAG, RVD, RC%, GTH, VAS-E, RES | RW (Baseline): Only significant difference between groups (p=0.033). KTH (3 months): CAF+L-PRF: 3.67 ± 0.488; CAF+A-PRF: 3.13 ± 0.640 (p=0.016*). MRC% (3 months): CAF+L-PRF: 67.20 ± 32.81%; CAF+A-PRF: 81.66 ± 28.21% (p=0.206). | At 3 months, the CAF + L-PRF group showed significantly higher KTH (p=0.016*). At 6 months, there was no statistically significant difference in clinical parameters. Although CAF + A-PRF had a higher MRC percentage at 6 months, it was not statistically significant compared to the CAF + L-PRF group. | Both treatment protocols (CAF+L-PRF and CAF+A-PRF) resulted in statistically significant improvements in all clinical parameters at 6 months. |
| Eren & Atilla [40] | Platelet-rich fibrin in the treatment of localized gingival,recessions: a split-mouth randomized clinical trial | 2014 | Turkey | Split-mouth, randomized, controlled, clinical trial | PRF (test). Combined with coronally advanced flap (CAF) | Gentle pressure for 3 minutes. Standardized photographs. | 6 months | 44 localized gingival recession defects (Miller I, II) in 22 patients | Not specified | Subepithelial Connective Tissue Graft (SCTG) | Yes. RD, RW, KTW, RA, PD, CAL, GT, % root coverage, % complete root coverage | Root coverage (%): Test: 92.7%; Control: 94.2% (p>0.05). Complete root coverage (%): Test: 72.7%; Control: 77.3% (p>0.05). KTW (6 months): Test: 3.51 ± 1.28 mm; Control: 3.63 ± 1.43 mm (p<0.001 for both). GT (6 months): Test: 1.59 ± 0.53 mm; Control: 1.68 ± 0.57 mm (p<0.001 for both). PD (6 months): Test: 1.09 ± 0.29 mm; Control: 1.45 ± 0.60 mm (p=0.017*). | There was no statistically significant difference in the percentage of root coverage, complete root coverage, KTW, and GT between the groups. The control group showed an increase in PD. | Both techniques (CAF+PRF and CAF+SCTG) are effective. PRF can be suggested as an alternative to SCTG due to the avoidance of a donor site and decreased patient discomfort. |
| Öngöz Dede et al. [41] | Comparison of the clinical efficacy of concentrated growth factor,and advanced platelet‐rich fibrin in the treatment of type I multiple,gingival recessions: a controlled randomized clinical trial | 2023 | Turkey | Randomized, parallel, controlled clinical trial | Concentrated Growth Factor (CGF) + CAF, A-PRF + CAF, or CAF alone | Oral hygiene instructions and full-mouth scaling 1 month before surgery. Instructions on non-traumatic brushing technique. | 6 months | 45 recessions with RT1 in 16 patients (multiple type I gingival recessions) | Not specified | A-PRF + CAF and CAF alone | Yes. PPD, CAL, VGR, KGW, GT, CRC, MRC | Not provided in the excerpts, only mention that 45 sites with defects completed the study without complications. | Not provided in the excerpts. | The objective is to compare the clinical effects of CGF + CAF, A-PRF + CAF, and CAF alone. |
| Barakat et al.[42] | Evaluation of advanced platelet‐rich fibrin compared to subepithelial,connective tissue graft in the surgical management of interdental,papilla recession: a randomized controlled trial | 2024 | Egypt | Prospective randomized controlled clinical trial | Multilayered advanced platelet-rich fibrin (A-PRF) membrane (experimental) | Not specified in the excerpts. | 1, 3, 6, 9, and 12 months | 32 deficient interdental papillae (IDP) with PPI of 2 or 3 | Not specified | Connective Tissue Graft (CTG) (control). Both with the Han and Takei approach | Yes. PH, % GBT change, postoperative pain (VAS), analgesic consumption | Mean gain in IDP height (12 months): CTG: 2.25 ± 0.97 mm; A-PRF: 1.86 ± 0.7 mm (non-significant difference). GBT fill (12 months): CTG: 57.98%; A-PRF: 54.65% (non-significant difference, p=0.956). Analgesic consumption: CTG: 11.75 ± 3.51; A-PRF: 8 ± 3.08 (p=0.003*). | Both CTG and A-PRF were equally effective in increasing IDP height and filling the GBT, with no significant difference. The CTG group consumed significantly more analgesics (p=0.003*) and reported more pain at the donor site. | A-PRF and CTG were equally effective. The multilayered A-PRF membrane appears to be a viable alternative to CTG in the treatment of GBTs. |
| Oliveira Alves et al. [43] | Comparative evaluation of effect of injectable platelet-rich fibrin with,collagen membrane compared with collagen membrane alone for,gingival recession coverage | 2025 | Brazil | Split-mouth, randomized controlled | Injectable I-PRF (experimental). Combined with scaling and root planing (SRP) | One injection of the adjunctive therapy at the end of the second SRP session. | 45 and 90 days | 24 patients (with two pairs of contralateral teeth with PD ≥ 5 mm). Stage III Grade B Periodontitis | Maxilla | SRP alone (control) | Yes. PD, GR, CAL, PI, fBOP, CEJ-BC | PD (90 days): SRP: 4.56 ± 1.42; SRP+i-PRF: 4.7 ± 1.5. GR (90 days): SRP: 0.5 ± 1.0; SRP+i-PRF: 0.6 ± 1.1. | Both groups showed significant reductions in PD, CAL, fBOP, and PI from baseline to 45 days (p < 0.001), with no significant differences between groups (p > 0.05). The test group exhibited an increase in GR at 45 days (p < 0.05). CEJ-BC decreased in both groups at 90 days with no significant intergroup difference (p > 0.05). | SRP alone or combined with i-PRF resulted in comparable clinical and radiographic improvements. Adjuvant i-PRF did not offer significant additional benefits. |

Source: Authors 2025
